# High frequency of SEN virus infection in thalassemic patients and healthy blood donors in Iran

**DOI:** 10.1186/1743-422X-7-1

**Published:** 2010-01-02

**Authors:** Abbas Karimi-Rastehkenari, Majid Bouzari

**Affiliations:** 1 Department of Biology, Faculty of Science, University of Isfahan, Hezar-jreeb Street, Postal code: 81746-73441, Isfahan, Iran

## Abstract

**Background:**

SEN virus is a blood-borne, circular ssDNA virus and possessing nine genotypes (A to I). Among nine genotypes, SENV-D and SENV-H genotypes have the strong link with patients with unknown (none-A to E) hepatitis infections. Infection with blood-borne viruses is the second important cause of death in thalassemic patients. The aim of this study was to determine the frequency of SENV-D and SENV-H genotypes viremia by performing nested-PCR in 120 and 100 sera from healthy blood donors and thalassemic patients in Guilan Province, North of Iran respectively. Also, to explicate a possible role of SEN virus in liver disease and established changes in blood factors, the serum aminotransferases (ALT and AST) and some of the blood factors were measured.

**Results:**

Frequency of SENV-D, SENV (SENV-H or SENV-D) and co-infection (both SENV-D and SENV-H) viremia was significantly higher among thalassemic patients than healthy individuals. Frequency of SENV-H viremia was significantly higher than SENV-D among healthy individuals. In comparison to SENV-D negative patients, the mean of mean corpuscular hemoglobin was significantly higher in SENV-D positive and co-infection cases (*P *< 0.05). The means of AST and ALT were significantly higher in thalassemic patients than healthy blood donors, but there were not any significant differences in the means of the liver levels between SENV-positive and -negative individuals in healthy blood donors and thalassemic patients. High nucleotide homology observed among PCR amplicon's sequences in healthy blood donors and thalassemic patients.

**Conclusions:**

The high rate of co-infection shows that different genotypes of SENV have no negative effects on each other. The high frequency of SENV infection among thalassemic patients suggests blood transfusion as main route of transmission. High frequency of SENV infection in healthy individuals indicates that other routes rather than blood transfusion also are important. Frequency of 90.8% of SENV infection among healthy blood donors as well as high nucleotide homology of sequenced amplicons between two groups can probably suggest that healthy blood donors infected by SENV act partly as a source of SENV transmission to the thalassemic patients. In conclusion, SENV-D isolate in Guilan Province may be having a pathogenic agent for thalassemic patients.

## Background

On July 20, 1999, SEN virus (SENV) was discovered in the serum of a human immunodeficiency virus type 1 (HIV-1) - infected patient possessing hepatitis with unknown etiology in Italy [1]. SENV is a blood-borne, circular ssDNA virus, with approximately 3800 nucleotides in length and about 26 nm in size that is non-enveloped and possesses at least 3 ORFs [2,3]. In the base of studies on ORF1 sequences SENV has been classified in a floating genus named Anellovirus [1,2]. Nine different genotypes (A to I) with at least 25% divergence in nucleotide sequence is reported [2,4]. Among nine genotypes, SENV-D and SENV-H genotypes have comparatively higher frequency in the patients with unknown (none-A to E) hepatitis and lower frequency in the sera of healthy blood donors [5]. It has also been shown that this virus is prevalent globally with various prevalence in different geographical areas [6].

Thalassemia is distributed widely in the Mediterranean area, Middle East, tropical Africa and the Caribbean [7]. After iron overload, blood-borne infections are the main cause of death in thalassemic patients [8].

The aim of this study was to determine the frequency of SENV-D and SENV-H genotypes viremia in thalassemic patients with high risk viremia for blood-borne viruses and healthy blood donors with low risk viremia for blood-borne viruses negative for HBs antigen, anti-HCV antiboby, anti-HIV antibody in Guilan Province, North of Iran.

Also, to explicate a possible role of SEN virus in liver disease and established changes in blood factors, the serum aminotransferases (ALT and AST) and some of the blood factors were measured.

## Methods

### Study design

Iran is located in world thalassemia belt with more than 25000 patients [9]. The Guilan Province lies along the south coast of Caspian Sea which seems the high rate of close relative marriage in this area, is the cause of high frequency of thalassemic patients. The sera were collected from 100 patients with thalassemia major from pathobiology laboratory of Razi Hospital in Rasht city from February to June, 2008 and 120 sera of healthy blood donors from blood transfusion organization of Guilan Province in September 2007 and stored in -20°C till tested. The serum samples were negative with ELISA tests for detection of HBs antigen (Dade Behring, Germany), anti-HCV antibody (Biomerieux, France) and HIV antigen-antibody (Bio Rad, France). Serum aminotransferases (AST and ALT) were measured by Man kit (Man laboratory, Iran). The blood factors including red blood cell count (RBC), white blood cell count (WBC), platelet count, hemoglobin (Hb), Hematocrit (HTC), mean corpuscular hemoglobin (MCH), mean corpuscular volume (MCV) and mean corpuscular hemoglobin concentration (MCHC) were measured according to the standard procedures.

### DNA extraction from serum

Serum (220 μl) was mixed with 10 μl of 0.2 M NaCl and 6.5 μl of 0.25% SDS. Twelve μl of 10 mg/ml proteinase K solution (Roche, Germany) was added and incubated at 65°C for 2 hours. Protein was precipitated with two phenol-chloroform and followed by only chloroform treatment. The cold ethanol (100%) (Merck, Germany) was used for DNA precipitation and the precipitate was dissolved in 50 μl of distilled dionized water.

### Detection of SENV DNA

Partial ORF1 gene of SENV-D and SENV-H were amplified by nested-PCR, with forward primer AI-1F (5'-TWC YCM AAC GAC CAG CTA GAC CT-3'; W = A or T, Y = C or T, M = A or C) and reverse primer AI-1R (5'-GTT TGT GGT GAG CAG AAC GGA-3') [4], for first round for all of the SENV genotypes. Master mix was made in a 25 μl volume with 0.4 pmol/μl of each primers, 50 mM of KCl, 20 mM Tris-HCl, 3 mM MgCl_2_, 240 μM of each dNTPs, 1 U of Smar *Taq *DNA polymerase (Cinnagen, Iran) and 3 μl of extracted DNA. Setting was 44 cycles (94°C for 20 seconds, 56°C for 25 seconds and 72°C for 30 seconds for each cycle) with a final extension time for 5 minutes at 72°C in a thermocycler gradient 5331 (Eppendorf, Germany). One microliter of the products of first-round PCR was used for the second-round PCR amplification with specific forward and reverse primers for SENV-D including D-1148F (5'-CTA AGC AGC CCT AAC ACT CAT CCA G-3') and D-1341R (5'-GCA GTT GAC CGC AAA GTT ACA AGA G-3') [4], and for SENV-H including H-1020F (5'-TTT GGC TGC ACC TTC TGG TT-3') and H-1138R (5'-AGA AAT GAT GGG TGA GTG TTA GGG-3') [4]. The second-round PCR involved 25 cycles (94°C for 20 seconds, 65°C for 30 seconds and 72°C for 30 seconds) for both SENV-D and SENV-H.

### DNA Sequencing

PCR products of four randomly selected samples from thalassemic patients and healthy blood donors were subjected to agarose gel electrophoresis (1.5%) and DNA was extracted according to guidelines of the DNA Gel Extraction Kit #K0513 (Fermentas, EU). The DNAs were sequenced by Geneservice Company, UK.

### Molecular evolutionary analyses

The sequences of the PCR amplicons were aligned using WU-BLAST2 method. Multiple alignments for the sequenced amplicons were performed with ClustalW in MEGA4 (Molecular Evolutionary Genetics Analysis software version 4.1) [10]. A phylogenetic tree constructed using neighbor-joining method based on partial ORF1 of our sequenced amplicons against sequences obtained from GenBank with accession numbers of GQ179968 and GQ179969 for SENV-D, and accession numbers of GQ179972 and GQ452051 for SENV-H, for healthy individuals and thalassemic patients, respectively. Eight SEN virus isolates (SENV-A to H), five TTV isolates and three variants of PMV, SANBAN and TLMV obtained from GenBank database.

### Statistical analyses

Fisher's exact test, unpaired *t*-test, one-way analysis of variance (ANOVA) and Tukey-Karmer post test were used for statistical analyses using the GraphPad Instat software version 3.05 (GraphPad software, USA) and SPSS software version 15.0 (SPSS Inc., USA).

## Results

**Figure 1:**
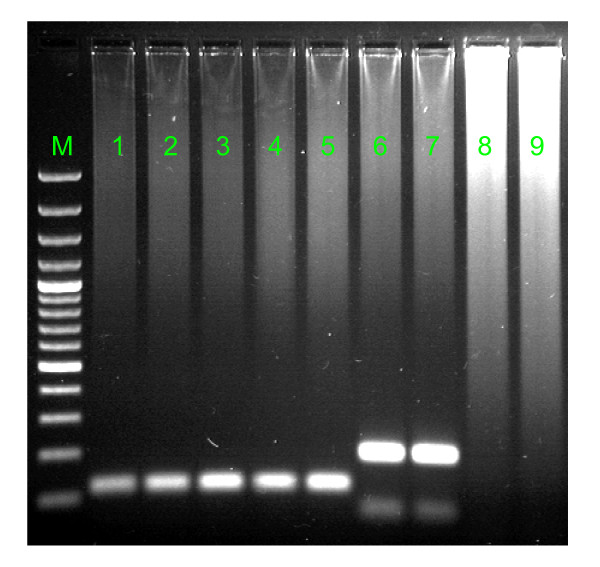
**Agarose gel electrophoresis of PCR products**. M: Marker 100 bp DNA (Fermentas, EU); columns 1-5 SENV-H positive (119 bp); columns 6 and 7 SENV-D positive (195 bp); columns 8 and 9 negative samples.

In the gel electrophoresis expected 195 bp bands for SENV-D and 119 bp bands for SENV-H were observed (Figure 1).

**Figure 2:**
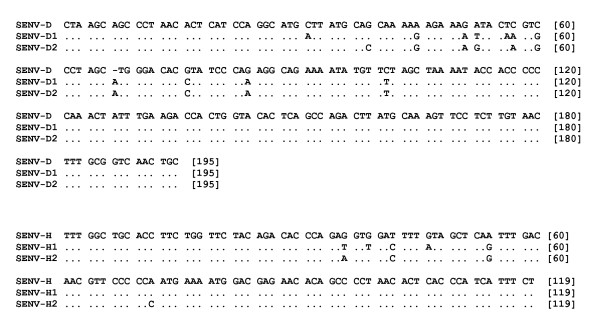
**Multiple alignments of PCR amplicons**. Multiple alignments of sequenced DNAs with accession numbers of [GenBank:GQ179968] and [GenBank:GQ179969] for SENV-D1 and SENV-D2, [GenBank:GQ179972] and [GenBank:GQ452051] for SENV-H1 and SENV-H2, respectively. Accession number of AX025730 for SENV-D and AX025838 for SENV-H obtained form GenBank. Only the nucleotides differed are shown. A gap was observed in location number 67 within SENV-D sequence.

As shown in figure 2, the homology was 98% between sequences of SENV-D1 [GenBank:GQ179968] and SENV-D2 [GenBank:GQ179969] sequences, likewise, the homology was 97% between SENV-H1 [GenBank:GQ179972] and SENV-H2 [GenBank:GQ452051] from Guilan isolates. Insertion of an adenine nucleotide in location number 67 was observed in multiple alignments of SENV-D1 and SENV-D2 sequences (in comparison to sequence with accession number AX025730).

**Figure 3:**
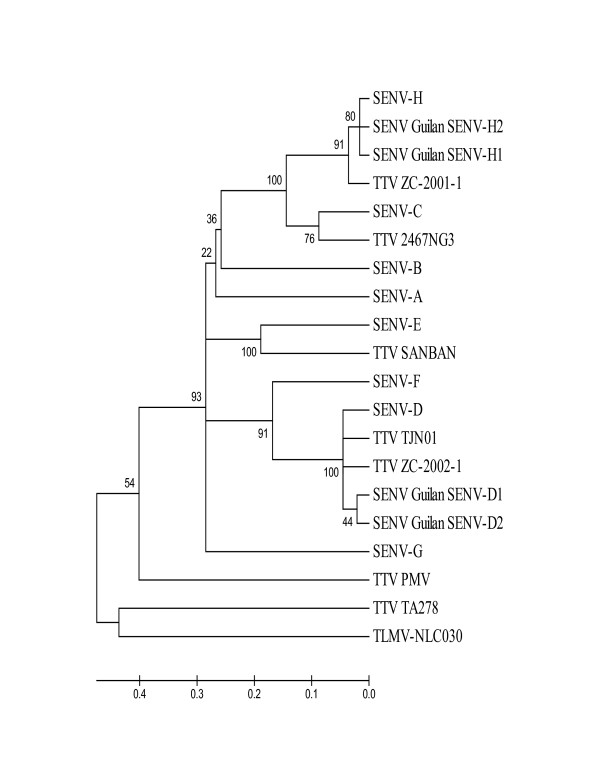
**Phylogenetic tree constructed by neighbor-joining method within partial ORF1 with 100 Bootstrap replicates**. Our sequences with accession numbers of GQ179968 and GQ179969 for SENV-D, and accession numbers of GQ179972 and GQ452051 for SENV-H, for healthy individuals and thalassemic patients, respectively. These 16 isolates comprise eight SEN virus isolates (SENV-A(AX025667), SENV-B(AX025677), SENV-C(AX025718), SENV-D(AX025730), SENV-E(AX025761), SENV-F(AX025822), SENV-G(AX025830), SENV-H(AX025838), and Five TT virus isolates (TA278(AB017610), TJN01(AB028668), ZC-2002-1(FM881988), 2467NG3(AY093401), ZC-2001-1(FM882007), and tree TTV variants PMV(AF261761), SANBAN(AB025946), TLMV(AB038631) obtained GenBank databases on NCBI website. The evolutionary distances were computed using the Maximum Composite Likelihood model based on the units of the number of base substitutions per site.

As shown in figure 3, high genomic homology observed between our sequences and some of the TTV isolates.

The hematological data of thalassemic patients are shown in table 1. Apart from three variables of MCH, WBC and platelet count, the rest were in normal range.

**Table 1:** Hematological data of thalassemic patients.

Gender (n = 100)	Age (year)	RBC (mil/mm^3^)	Hb (gr/dl)	HCT (%)	MCV (fl)	MCHC (gr/dl)
Female (n = 49)	23.4 ± 1.9	3.2 ± 0.1	8.4 ± 0.3	26.7 ± 1.0	83.3 ± 1.2	31.4 ± 0.4
Male (n = 51)	23.2 ± 1.4	3.1 ± 0.0	8.3 ± 0.2	26.7 ± 0.9	84.6 ± 1.4	31.07 ± 0.3

Data expressed as mean ± SD; RBC, red blood cell count; Hb, hemoglobin; HTC, Hematocrit; MCV, mean corpuscular volume; MCHC, mean corpuscular hemoglobin concentration.

The comparison of age, gender and paraclinical characteristics of the thalassemic patients and healthy blood donors are shown in table 2. The mean age and frequency of males were significantly higher in healthy blood donors (*P *< 0.0001). Conversely, the means of AST and ALT were significantly higher in thalassemic patients (*P *< 0.001).

**Table 2:** Comparison of paraclinical characteristics of thalassemic patients and healthy blood donors.

Paraclinical characteristics	Healthy blood donors (N = 120)	Thalassemic patients (N = 100)	*P *value
Age (years)	35.2 ± 9.4	22.4 ± 6.1	<0.0001
Gender (% male)	110 (91.6)	49 (49)	<0.0001
AST† (IU/L)	14.9 ± 15.3	27.6 ± 18.6	<0.001
ALT† (IU/L)	9.4 ± 10.1	25.7 ± 19.1	<0.001

† Normal range, 0-46 IU/L; AST, Aspartate aminotransferase; ALT, Alanine aminotransferase.

**Figure 4:**
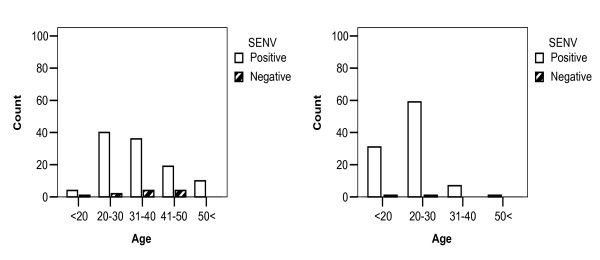
**Comparison of correlation between age groups and SENV-infected and SENV-uninfected individuals in healthy individuals (left), and thalassemic patients (right)**.

Comparison of correlation between age groups and individuals with SENV-positive versus SENV-negative viremia in healthy blood donors and thalassemic patients are shown in figure 4. Forty percent of SENV-positive healthy blood donors were under 30 years, while this was 91% in thalassemic patients which mostly trends to younger age group.

Frequency of SENV-D, SENV and co-infection viremia was significantly higher among thalassemic patients than healthy blood donors. Conversely, there was no significant difference in the frequency of SENV-H between healthy blood donors and thalassemic patients. Furthermore, frequency of SENV-H viremia was significantly higher than SENV-D among healthy blood donors, while this was not significant in thalassemic patients (Table 3).

**Table 3:** Frequency of SEN virus infection among thalassemic patients and healthy blood donors.

Virus viremia	Healthy blood donors (N = 120)	Thalassemic patients (N = 100)	*P *value	Odd ratio (95% CI)
SENV-D (+) [N (%)]	73 (60.8%)	86 (86%)	<0.0001	0.25 (0.12-0.49)
SENV-H (+) [N (%)]	103 (85.8%)	93 (93%)	0.12	0.45 (0.18-1.14)
† Co-infection (+) [N (%)]	67 (55.8%)	81 (81%)	<0.0001	0.29 (0.16-0.54)
SENV (+) [N (%)]	109 (90.8%)	98 (98%)	0.040	0.20 (0.04-0.93)

† Co-infection, SENV-D and SENV-H; Fisher's exact test.

The comparison of paraclinical characteristics in thalassemic patients and healthy blood donors with and without SENV infection are shown in tables 4 and 5.

**Table 4:** Comparison of paraclinical characteristics in thalassemic patients with and without SENV infection.

	SENV		SENV-D		SENV-H		Co-infection(+)
				
Characteristics	+ (N = 98)	- (N = 2)	+ (N = 86)	- (N = 14)	+ (N = 93)	- (N = 7)	(N = 81)
Gender [male (%)]	48 (49%)	1 (50%)	41 (47%)	8 (57%)	46 (49%)	3 (42%)	39 (48%)
Age (years)	22.4 ± 6.2	20.5 ± 2.1	22.2 ± 5.5	23.8 ± 9.3	22.4 ± 6.3	22.6 ± 4.0	22.1 ± 5.5
WBC count (× 10^3^)	14.9 ± 15.9	7.7 ± 2.7	14.7 ± 14.7	15.0 ± 21.9	14.1 ± 15.1	23.8 ± 23.0	13.8 ± 13.6
Platelet count (× 10^4^)	43.9 ± 25.5	32.8 ± 18.1	45.3 ± 26.2	33.7 ± 16.2	43.6 ± 25.6	44.2 ± 23.0	45.1 ± 26.4
MCH (pg)	26.1 ± 1.4	24.7 ± 1.1	26.212 ± 1.390†	25.321 ± 1.632	26.1 ± 1.5	25.9 ± 1.5	26.201 ± 1.394‡
ALT (IU/L)	26.0 ± 19.1	13.5 ± 14.2	26.7 ± 19.2	18.1 ± 17.8	25.7 ± 19.1	22.9 ± 21.9	26.5 ± 19.2
AST (IU/L)	27.7 ± 18.7	21.4 ± 13.0	27.6 ± 18.5	27.8 ± 19.5	27.7 ± 18.9	26.1 ± 15.0	27.5 ± 18.7

†*P *= 0.032 for SENV-D (+) vs. SENV-D (-), ‡*P *= 0.036 for Co-infection (+) vs. SENV-D (-)

Normal range for platelet count (15-40 × 10^4^/mm^3^); WBC, white blood cell count (4-10 × 10^3^/mm^3^) and MCH, mean corpuscular hemoglobin (26-33 pg).

**Table 5:** Comparison of paraclinical characteristics of healthy blood donors with and without SENV infection.

Characteristics	SENV(-) (N = 11)	SENV(+) (N = 109)	SENV-D(+) (N = 73)	SENV-H(+) (N = 103)	Co-infection(+) (N = 67)
Gender [Male]	11	98	63	95	59
Age (years)	34.1 ± 8.1	35.3 ± 9.6	36.2 ± 10.2	35.4 ± 9.6	36.3 ± 10.4
ALT (IU/L)	9.8 ± 11.0	9.4 ± 10.1	8.5 ± 8.9	9.3 ± 10.1	8.4 ± 8.9
AST (IU/L)	7.4 ± 9.0	15.6 ± 15.7	14.5 ± 14.5	15.7 ± 15.9	14.6 ± 14.7

† Normal range, 0-46 IU/L; AST, Aspartate aminotransferase; ALT, Alanine aminotransferase.

The differences of the white blood cell and platelet count of the patients were not significant (*P *> 0.05). In comparison to SENV-D negative patients the mean of MCH was significantly higher in SENV-D positive and co-infection cases (*P *< 0.05).

No significant differences were observed in the mean of age of individuals positive and negative for SENV, SENV-D, SENV-H and co-infection (Table 4).

There were not any significant differences in the mean of ALT and AST levels between SENV-positive and -negative individuals in healthy blood donors and thalassemic patients (Tables 4 and 5). It is notable that the amount of AST and ALT was higher than normal in twenty-six thalassemic patients.

**Figure 5:**
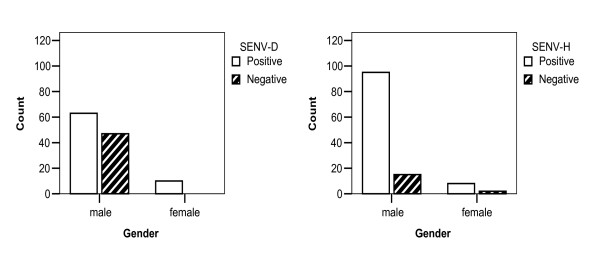
**Comparison of correlation between gender and frequency of SENV-D and SENV-H among healthy blood donors**.

As shown in Figure 5, SENV-H positive male individuals were significantly higher than SENV-D positive ones (*P *< 0.001).

## Discussion

Worldwide distribution of SENV is already reported in healthy blood donors from various geographic areas such as U.S.A (1.8%) [5], Japan (10-22%) [11], Taiwan (15%) [12], Thailand (5%) [13], Germany (8-17%) [14], and at least 13% in Italy [15].

The percentage of SENV infection in healthy blood donors in this study was 90.8% that is much higher than previous reports. On the other hand rarely it resembles to 75% of SENV infection reported in Japan by Yoshida et al. (2002) [16]. Wide ranges of infection is reported in intravenous drug users, hemophilic and thalassemic patients, patients on maintenance hemodialysis, HIV positive and individuals with liver disease [12,17,18]. Ninety-eight percent of SENV infection in thalassemic patients is similar to the results obtained in Taiwan (90%) but in healthy individuals tested it was 90.8% versus 15% in Taiwan [12].

In comparison to other areas studied, the higher frequency of SENV infection in our study could be correlated to the methods used. Higher percentage (90.8%) of SENV infection in North of Iran, in comparison to other healthy blood donors in center of Iran (Tehran Province) (23%) [19], can probably be due to differences in the methods used and climate conditions including temperate and humid climate in Guilan Province against Tehran which is warm and dry. This might affect the durability of SENV in the environment.

In three separate investigations on interferon and combination therapy of SENV, it is shown that in comparison to SENV-H, SENV-D is more susceptible to the interferon therapy [20-22]. The lower frequency of SENV-D observed in this study might be correlated to possible primary interferon response. It is shown that SENV can be transmitted vertically [23,24]. According to Kao et al. (2002) [12], and Serin et al. findings (2005) [25], the prevalence of SENV in patients with acute hepatitis A infection is higher than healthy individuals. They proposed the fecal-oral transmission route for SENV.

Although no significant correlation was observed in the level of ALT and AST in healthy blood donors and thalassemic patient with or without SENV infection, 26 thalassemic patients showed unnormal upper levels of the enzymes (46 IU/L). SENV-D viremia had significant effects on the MCH of the thalassemic patients (*P *< .05). It is already reported that the SENV has an adverse effect on the survival of the HIV-positive patients (Sagir et al., 2005) [26]. According to the Figure 4, the effect of SENV on the survival of thalassemic patients remained unknown.

High genomic homology observed between our sequences and some of the TTV isolates may be the outlook to the evolutionary history of SENV in relation to TTV as already expressed by Tanaka et al. (2001) [2].

Our results demonstrates that the frequency of SENV-H is higher than SENV-D among healthy blood donors that is consistent with Kao et al. findings (2002) [12].

Considering the reports of the replication of the virus in liver cells and the failure of manifesting clinical signs in infections such as cytomegalovirus, Epstein-Barr, Hepatitis A and B is common in immunocompetent individuals [5], the high frequency of SENV in healthy blood donors with no liver malfunction is a vague result.

## Conclusions

The high rate of co-infection shows that different genotypes of the virus have no negative effects on each other.

Higher frequency of SENV infection among thalassemic patients in comparison to healthy blood donors, except for nearly identical frequency of SENV-H in healthy blood donors and thalassemic patients (no significant difference), indicates the main route of blood trnasfusion. The high frequency of SENV infection among healthy blood donors suggests that SENV is also transmitted by different routes rather than blood transfusion route.

According to the Tanaka et al. findings, some of the TTV-related isolates can be pathogenic [2]. Considering to the obtained results, SENV-D isolate in Guilan Province may be pathogenic for thalassemic patients.

Frequency of 90.8% of SENV infection among healthy blood donors as well as high nucleotide homology of sequenced amplicons between two groups can probably suggest that healthy blood donors infected by SENV act partly as a source of SENV transmission to the thalassemic patients and possibly to other community groups.

## List of abbreviations

SENV-D: SEN virus genotype D; SENV-H: SEN virus genotype H; TTV: TT virus; PCR: polymerase chain reaction; MCH: mean corpuscular hemoglobin.

## Competing interests

The authors declare that they have no competing interests.

## Authors' contributions

MB performed the design of the study, designed the genetical and statistical analyses, supervised and co-wrote the manuscript. AK-R performed the experimental work and genetical and statistical analyses, collected the sera and data, interpreted the results and drafted primary version of the manuscript.

Both authors read and approved the final manuscript.
